# Headache in the Emergency Department: A Multicenter Observational Study from Singapore

**DOI:** 10.3390/medicina59071340

**Published:** 2023-07-21

**Authors:** Win Sen Kuan, Ranjeev Kumar, Ying Wei Yau, Wei Ming Ng, Dennis Wen Jie Chia, Ee Yang Ng, Kanwar Sudhir Lather, Mui Teng Chua

**Affiliations:** 1 Emergency Medicine Department, National University Hospital, National University Health System, Singapore 119074, Singapore; ying_wei_yau@nuhs.edu.sg (Y.W.Y.); kanwar_sudhir_lather@nuhs.edu.sg (K.S.L.); mui_teng_chua@nuhs.edu.sg (M.T.C.); 2 Department of Surgery, Yong Loo Lin School of Medicine, National University of Singapore, Singapore 119228, Singapore; 3 Acute & Emergency Care Centre, Khoo Teck Puat Hospital, National Healthcare Group, Singapore 768828, Singapore; ranjeev.kumar@ktph.com.sg (R.K.); ng.ee.yang@ktph.com.sg (E.Y.N.); 4 Emergency Department, Ng Teng Fong General Hospital, National University Health System, Singapore 609606, Singapore; wei_ming_ng@nuhs.edu.sg; 5 Emergency Department, Sengkang General Hospital, Singapore Health Services, Singapore 544886, Singapore; dennis.chia.w.j@singhealth.com.sg

**Keywords:** emergency department, epidemiology, headache, opioid

## Abstract

*Background and Objectives*: There is scarce data about the epidemiology, clinical features, investigations, diagnosis, treatment, and outcome in patients attending Singapore emergency departments (EDs) with nontraumatic headache. We sought to describe these characteristics of adult patients presenting to the ED with a primary complaint of headache. *Materials and Methods*: We performed a cross-sectional study on adult patients with nontraumatic headache over 4 consecutive weeks from 18 March 2019 to 14 April 2019 across four EDs in Singapore. Exclusion criteria were history of head trauma within 48 h of presentation, missing records, interhospital transfers, representation with the same headache as a recent previous visit and headache as an associated symptom. *Results*: During the study period, 579 patients (representing 1.8% of the total ED census) comprising 55.3% males and with a median age of 36 years presented to the four Singapore EDs with a primary complaint of nontraumatic headache. Paracetamol (41.5%), non-steroidal anti-inflammatory drugs (34.4%) and tramadol (31.5%) were the three commonest analgesics used either singly or in combination. Prochlorperazine (22.9%) and metoclopramide (17.4%) were frequent anti-emetic adjuncts. One-third of patients had computed tomography of the brain performed, which found abnormalities among 20.9% of them. ED diagnoses of primary headache conditions were made in 73.6% of patients. *Conclusions*: Primary headaches constituted most ED headache diagnoses. ED imaging of selected patients yielded a relatively high pick-up rate for significant intracranial abnormalities. Opioid use for symptomatic relief of headaches in the ED was found to be high, underscoring the need for improvement in headache analgesia relief practices in the ED.

## 1. Introduction

Headache disorders constitute a high global burden of disease [1]. Being a prevalent and disabling condition, headache is one of the commonest presenting complaints in the emergency department (ED), accounting for about 3% of all ED visits in the US [2]. In a Singapore community-based study, the overall lifetime prevalence of headache was 82.7% [3]. Another one-year single center study in Singapore showed that nontraumatic headache contributed to 2% of ED attendances and over 17% of them were subsequently admitted [4].

There are many varying types and underlying etiologies of primary and secondary headaches. Although most ED patients have benign primary headaches (e.g., migraine, tension-type, cluster), differentiating from emergency and life-threatening conditions like hemorrhage or ischemic strokes, intracranial infections, tumors, and toxicities are of the utmost priority in the ED [5]. Several national clinical policies and guidelines for assessment and treatment of headache in the ED are available [6,7,8,9]. Despite this, there exists wide variation in practice among emergency physicians [10]. It is unknown whether this variation stems from clinician, institution, or regional sociocultural factors, or whether it is due to lack of evidence regarding effective treatment options. Unmet needs on this topic in the undergraduate curriculum may also be present, which could partially explain the lack of standardization in practice [11].

In Singapore, data regarding the epidemiology of nontraumatic headache in patients attending EDs are scanty. Hence, this study aimed to describe the clinical features, investigations, diagnosis, treatment, and outcome of nontraumatic headache in adults who present to EDs in Singapore with headache as their primary complaint, and to compare the similarities and differences of these characteristics among institutions.

## 2. Materials and Methods

This was a planned sub-study of a multicenter observational cross-sectional study conducted over 4 consecutive weeks from 18 March 2019 to 14 April 2019. Institutions involved were National University Hospital (NUH), a tertiary academic medical center, and 3 general hospitals—Khoo Teck Puat Hospital (KTPH), Ng Teng Fong General Hospital (NTFGH) and Sengkang General Hospital (SKH). Details of the parent study, Headache in Emergency Departments (HEAD study), is described elsewhere [12]. Briefly, it included participants who were adult patients (aged 21 years and above in Singapore) with nontraumatic headache as their presenting complaint. Exclusion criteria were history of trauma to the head within 48 h of presentation, missing records, interhospital transfers, recurring presentation with the same headache as a recent previous visit and headache as an associated symptom rather than a main complaint.

Determination of whether headache was a primary complaint was at the discretion of the site investigators based on all available data. Eligible adult patients presenting during the study period were identified from the respective institution’s ED data management system. Data were collected retrospectively and included demographics, clinical assessment, investigation, diagnosis, treatment, disposition, and outcome. Data were entered onto piloted data forms or directly into the study database depending on institutional processes and resources. Study data were collected and managed using REDCap (Research Electronic Data Capture) hosted at the Joseph Epstein Centre for Emergency Medicine Research, Melbourne, Australia [13]. Outcomes of interest for this study include demographics, clinical features, patterns of investigation, treatment, disposition, and outcome of patients presenting with headache to the 4 participating EDs in Singapore.

### Statistical Analyses

Statistical analyses were carried out using Stata version 15 (College Station, TX, USA). Data analyses were predominantly descriptive. Categorical variables are reported in proportions while continuous variables are reported in median with interquartile range (IQR). Differences in categorical variables were compared with chi-squared test or Fisher’s exact test, while differences in continuous variables were compared using Kruskal–Wallis test. Ethics approval was obtained from the National Healthcare Group Domain Specific Review Board (DSRB 2018/01052) who granted waiver of informed consent. The parent study was registered with the Australia and New Zealand Clinical Trials Registry (trial number 376695).

## 3. Results

Four Singapore institutions that were included in the study had a combined ED census of 32,425 adult patients between 18 March 2019 and 14 April 2019 (Supplementary Table S1). A total of 579 (1.8%) patients presented to these 4 EDs with a main complaint of nontraumatic headache during the 4-week study period.

### 3.1. Patient Characteristics

The median age was 36 (IQR 26 to 51) years with a predominance of male patients (320/579, 55.3%) (Table 1). Overall, the majority (332/579, 57.3%) of patients had symptoms for 3 days or fewer prior to ED attendance. Only 3.6% (21/579) were delivered by ambulance. About one quarter (147/579, 25.4%) had presence of preexisting conditions potentially related to the presenting headache episode and 9.0% (52/579) were on regular medications for their headache.

Clinical features including symptoms and signs are summarized in Table 2. Sudden or “thunderclap” onset of headache was documented in 6.2% (36/579) of patients. Just under half (267/579, 46.1%) of the patients reported moderate severity of their headache. Nausea or vomiting were the most predominant (200/579, 34.5%) associated symptoms, followed by neck pain or stiffness (71/579, 12.3%) and new visual disturbance (56/579, 9.7%). A small proportion (9/457, 2.0%) had a documented Glasgow Coma Score of below 15. Among 24.0% (139/579) of patients who had pre-medicated before ED attendance, paracetamol (110/139, 79.1%) and non-steroidal anti-inflammatory drugs (NSAIDs) (26/139, 18.7%) were the medications most often taken for pain relief prior to ED attendance.

### 3.2. Investigations and Treatment

One-third (191/579, 33.0%) of patients had computed tomography (CT) brain performed, which yielded abnormalities in 20.9% (40/191) (Table 3). Among the abnormal CT brain findings, there were 11 patients who had intracranial hemorrhages, including one with subarachnoid hemorrhage (SAH). One patient had lumbar puncture performed in the ED during the study period.

A total of 340 (58.7%) patients received medications in the ED. Paracetamol (41.5%), NSAIDs (34.4%) and tramadol (31.5%) were the three most common analgesics used either singly or in combination. Prochlorperazine (22.9%) and metoclopramide (17.4%) were frequent anti-emetic adjuncts. A smaller proportion (49/340, 14.5%) required further follow-up medications after 30 min from the first dose.

### 3.3. Main Diagnosis and Outcomes

Almost three-quarters (426/579, 73.6%) of patients who presented with nontraumatic headache as their primary complaint were diagnosed to have primary headache (unspecified primary benign headache, migraine, tension headache and cluster headache) (Table 4 and Supplementary Table S2). Other causes of headache were attributed to sinusitis (4.3%), hypertension (2.8%), musculoskeletal headaches including cervicogenic origin (2.1%), viral illness including dengue fever (1.6%) and upper respiratory tract infection (1.4%). More serious causes of headache include intracranial hemorrhage, besides SAH (1.4%) and ischemic stroke (1.2%) (Supplementary Table S2). The cause of headache was still unclear in the ED in 8.6% (50/579) of patients.

The majority of patients were discharged directly from the ED (72.9%), or after up to a 24-h period of observation in the ED observation unit (3.5%). Fewer than one quarter (132/579, 22.8%) were admitted to the general ward. The median length of stay was 2 days. Following hospitalization, the causes of headache were most commonly attributed to migraine (24.3%), unspecified primary benign headache (17.5%), tension headache (14.6%) and hypertension (8.0%). Six unscheduled reattendances to the ED within 72 h (four patients had primary benign headaches and two patients had sinusitis) and two deaths (both from intracranial hemorrhages) occurred during the 4-week study period.

### 3.4. Comparison among Institutions

There was notable female predominance at NUH (59.8%) compared to the other institutions (*p* < 0.001) (Table 1). Significantly more patients in SKH had preexisting headache-related conditions (46.6%) and were on regular headache medications (23.3%) compared to other institutions (both *p* < 0.001). A higher proportion of patients were also triaged to the “urgent” category in NUH (45.7%) and SKH (50.5%) compared to the other two institutions. Of significance, the cause of the headache was deemed “unclear” in 36.9% of patients in SKH ED compared to 7.2% in NUH and none in KTPH and NTFGH.

CT brain was performed least frequently in NUH (25.6%) and most frequently in SKH (44.7%) (*p* = 0.011), though the proportion of abnormal findings were not statistically significantly different among all four institutions (*p* = 0.150) (Table 3). Magnetic resonance imaging of the brain was exclusively performed in SKH (nine patients) and NTFGH (two patients). Medications were given in the ED to 74.8% of patients in SKH compared to 53.3% to 57.1% in the other institutions (*p* = 0.003). Anti-emetic adjuncts of prochlorperazine (34.1%) and metoclopramide (28.6%) were most commonly prescribed in the EDs of NUH and NTFGH, respectively.

Patients with headache were more likely to be admitted in SKH (39.8%) and least likely to be admitted in KTPH (12.1%) (Table 4) in tandem with the overall admission rates in the EDs of SKH (40.0%) and KTPH (24.6%) (Supplementary Table S1).

## 4. Discussion

In our cohort of 579 patients with primary complaint of nontraumatic headache from four Singapore EDs, overall predominance of male patients contrasts with previous observations from the US [14], Australia [15] and Europe [16], as well as from the multinational parent HEAD study [12]. This could be related to the overall higher proportion of male patients (about 56%) who attend EDs in Singapore [17,18]. Despite this, there were more females (59/106, 55.7%) diagnosed with migraine in the ED, concurring with the international literature, though not quite at the three-fold level more commonly found in previous studies [19].

A large retrospective US study showed that patients aged over 50 years were four times more likely to have a pathologic diagnosis presenting as headache, such as intracranial hemorrhage, acute angle closure glaucoma, giant cell arteritis, and malignancy [14]. Our study only had 3.8% of patients who were over 50 years compared to the US cohort (18.8%) and HEAD study (32.0%). This was despite Singapore having a rapidly aging population, with citizens aged 65 and above comprising 16.0% of the population in 2019 [20]. The discrepancy is possibly attributable to yet to be determined healthcare system and socioeconomic factors, along with health seeking behavior pertaining to ED use in Singapore.

An alarmingly high proportion of patients with nontraumatic headache in Singapore received opioids for analgesia in the ED, predominantly in the form of tramadol (31.5%) and codeine (5.6%), even though only 2.9% of patients among those who pre-medicated prior to ED arrival took tramadol. The high use of opioid analgesia for headache is against well-established recommendations, as there is robust evidence supporting the use of multimodal, nonopioid analgesics in the ED [21]. Opioid use has been shown to increase the risk of medication over use for headaches [22]. In migraine, opioids have been recognized as ineffective, potentially habit forming, and inferior to nonopioid options [23]. A review of our study’s drug prescriptions appears to indicate a belief among emergency physicians that opioids are effective and appropriate for the acute treatment of primary headaches. The under-recognition of medication overuse for headache in our study (0.2%) substantiates the existing literature locally [24]. These findings highlight the need for identifying the root cause behind the opioid use for headaches in emergency physicians, and thereafter to devise strategies to reduce this practice gap [25].

Within the study period, brain CT was performed in 33.0% of patients, which is comparable to proportions described in other studies (33% to 53%) [25,26]. Forty (20.9% of scanned; 6.9% of whole cohort) patients showed significant intracranial abnormalities (e.g., intracranial bleeding, tumors, cerebrovascular disease). This diagnostic yield is higher than that mentioned in some reviewed literature (2.5% to 10%) [26,27]. However, similar positive findings (23.1%) were reported in a prospective single center study conducted in an ED cohort of 1132 patients in Switzerland [28]. We surmise that the high diagnostic yield could be a result of existence of protocols for case discussion with emergency medicine specialists prior to approval for CT scans, thereby allowing additional experienced clinical judgment in imaging decisions, in addition to decision making tools, possibly improving case selection for patients going for brain CT. The high overall incidence of significant intracranial abnormalities in our patients showed that the selection of patients to undergo neuroimaging was adequate.

There were 96 (16.6%) patients who presented with elevated blood pressure (BP) (systolic BP > 160 mmHg) in our study (Table 2). Interestingly, 16 (2.8%) patients from the ED, and 11 (8.0%) patients from the wards were given the discharge diagnosis of ‘hypertension’. Analysis of two large datasets in the US found that, while elevated BP is common among ED patients who present with a chief complaint of headache, ED patients with headache were more likely to have elevated BP than are ED patients with other chief complaints [29]. Among patients who present to an ED with migraine and an elevated BP, there is no correlation between improvement in headache and improvement in systolic or diastolic BP, therefore indicating that the presence of headache has no practical consequence for the emergent management of hypertension in the ED, except for hypertensive crisis associated with neurological signs suggestive of hypertensive encephalopathy. In an analysis of 1914 patients with 30-year follow-up, the presence of headache was not associated with worse outcome regarding all-cause and cardiovascular mortality [30]. Paradoxically, headache appeared to carry a protective effect, showing a decreased risk for all-cause mortality and cardiovascular mortality but not for stroke mortality—where the outcomes were similar between the headache and no-headache cohorts.

A significant proportion (44.4%) of headache diagnoses were entered as ‘primary headache, not otherwise specified’, without a more specific International Classification of Headache Disorder (ICHD) diagnosis [31]. This is comparable to the 44% reported by Chu et al. [15], but higher than the 36% reported by Friedman et al., who conducted detailed structured patient interviews with the assistance of trained research associates [32]. Accurate headache diagnosis is possibly useful in standardization, evaluation, or improvement in headache management. In the third edition of the ICHD, the presenting headache needs to meet specific criteria prior to being allocated an appropriate primary headache subtype diagnosis: specific number of prior occurrences; specific length of time; typical quality, location, and exacerbating factors; must have (or lack) characteristic associated symptoms; secondary headache disorders must be excluded as the true diagnosis. Such detailed history taken from a distressed patient may prove challenging in the busy ED.

The strength of this study is its generalizability—all patients presenting to the EDs were included, 24 h per day, by many clinicians, across all four institutions covering the southwest and northeast of Singapore serving approximately 2 million residents, which minimizes the risk of systematic selection bias. This is the first study to record real-world data on the patient demographics, clinical characteristics, management details and outcomes for over 500 nontraumatic headache presentations in adult patients across multiple EDs in Singapore.

There are several limitations of the study. First, the study was retrospective in nature with its inherent biases. Clinical data were collected by the treating physician and not by dedicated trained headache experts. The case report forms for included variables were fixed at the steering committee level, thus certain details of data for explanatory purposes may be lacking. Second, data collected during the 4-week period may be over too short a period and not representative of annual trends in the respective institutions; however, given the lack of seasonal variations in Singapore, this bias is likely minimal. Third, data was obtained from SKH just 3 months after its official opening, which may not be representative of its current state. Lastly, patient enrolment based on the doctors’ assessment that the headache was a primary symptom may have an element of subjectivity and could have led to an undercounting of serious illnesses where headache was an associated symptom.

## 5. Conclusions

Primary headaches comprised the overwhelming majority of ED headache diagnoses in Singapore, with migraine being the most frequent primary headache diagnosis. ED imaging of selected headache patients showed a relatively high pick-up rate for significant intracranial abnormalities. Opioid use for symptomatic relief of headaches in the ED was found to be high, incongruent with guidelines, thereby underscoring the need for improvement in headache analgesia relief practices in the ED.

## Figures and Tables

**Table 1:** Patient demographics.

Variables	Total (*n =* 579)	KTPH (*n =* 165)	NUH (*n =* 164)	NTFGH (*n =* 147)	SKH (*n =* 103)	*p* Value
Age in years, median (IQR)	36 (26–51)	29 (23–43)	40 (29–54)	37 (24–51)	38 (31–51)	<0.001 *
Age > 50 years	22 (3.8)	5 (3.0)	8 (4.9)	5 (3.4)	4 (3.9)	0.836
Male gender	320 (55.3)	111 (67.3)	66 (40.2)	91 (61.9)	52 (50.5)	<0.001
Duration of symptoms						
<24 h	161 (27.8)	54 (32.7)	36 (22.0)	42 (28.6)	29 (28.2)	
1–3 days	171 (29.5)	47 (28.5)	48 (29.3)	46 (31.3)	30 (29.1)	0.697
>3 days	231 (39.9)	59 (35.8)	75 (45.7)	55 (37.4)	42 (40.8)	
Unknown	16 (2.8)	5 (3.0)	5 (3.0)	4 (2.7)	2 (1.9)	
Presence of preexisting conditions potentially related to current headache episode ^a^	147 (25.4)	28 (17.0)	50 (30.5)	21 (14.3)	48 (46.6)	<0.001
Recurrent headache	26 (14.5)	3 (8.6)	7 (13.2)	4 (19.1)	12 (17.1)	0.614
Migraine	69 (38.6)	10 (28.6)	25 (47.2)	12 (57.1)	22 (31.4)	0.053
Tension headache	8 (4.5)	2 (5.7)	4 (7.6)	1 (4.8)	1 (1.4)	0.420
Previous stroke	18 (10.1)	9 (25.7)	4 (7.6)	0	5 (7.1)	0.005
Previous ICH	3 (1.7)	0	1 (1.9)	1 (4.8)	1 (1.4)	0.604
Malignancy ^b^/VP shunt/ intracranial hypertension	15 (8.4)	0	7 (13.2)	2 (9.5)	6 (8.6)	0.183
Previous aneurysm/AVM/SAH	2 (1.1)	1 (2.9)	1 (1.9)	0	0	0.518
Others	14 (7.8)	5 (14.3)	6 (11.3)	1 (4.8)	2 (2.9)	0.133
On regular medications for headache	52 (9.0)	16 (9.7)	10 (6.1)	2 (1.4)	24 (23.3)	<0.001
Referral by a doctor	114 (19.7)	30 (18.2)	32 (19.5)	24 (16.3)	28 (27.2)	0.175
Conveyed by ambulance	21 (3.6)	3 (1.8)	2 (1.2)	10 (6.8)	6 (5.8)	0.054
Triage category						
Immediate	6 (1.0)	2 (1.2)	2 (1.2)	2 (1.4)	0 (0.0)	
Urgent	201 (34.7)	28 (17.0)	75 (45.7)	46 (31.3)	52 (50.5)	<0.001
Non-urgent	372 (64.3)	135 (81.8)	87 (53.1)	99 (67.3)	51 (49.5)	

Data are reported as *n* (%) unless otherwise stated. AVM: arteriovenous malformation; ICH: intracranial hemorrhage; IQR: interquartile range; SAH: subarachnoid hemorrhage; VP: ventriculoperitoneal. All *p* Values obtained by chi-squared test unless otherwise stated. * Kruskal-Wallis test. ^a^ Some patients have more than one condition. ^b^ One patient had both VP shunt and malignancy.

**Table 2:** Clinical features.

Variables	Total (*n =* 579)	KTPH (*n =* 165)	NUH (*n =* 164)	NTFGH (*n =* 147)	SKH (*n =* 103)	*p* Value
History						
Onset of symptoms						
Gradual	200 (34.5)	18 (10.9)	92 (56.1)	24 (16.3)	66 (64.1)	
Sudden or thunderclap	36 (6.2)	13 (7.9)	10 (6.1)	8 (5.4)	5 (4.8)	<0.001
Peak intensity < 1 h	9 (1.6)	1 (0.6)	7 (4.3)	1 (0.7)	0 (0.0)	
Unknown	334 (57.7)	133 (80.6)	55 (33.5)	114 (77.6)	32 (31.1)	
Head trauma within last week	7 (1.2)	1 (0.6)	2 (1.2)	1 (0.7)	3 (2.9)	0.341
Location of headache						
Generalized	178 (30.7)	52 (31.5)	74 (45.1)	20 (13.6)	32 (31.1)	
Unilateral	240 (41.5)	70 (42.4)	61 (37.2)	50 (34.0)	59 (57.3)	<0.001
Unclear	161 (27.8)	43 (26.1)	29 (17.7)	77 (52.4)	12 (11.6)	
Severity of headache						
Mild	147 (25.4)	36 (21.8)	48 (29.3)	41 (27.9)	22 (21.4)	
Moderate	267 (46.1)	88 (53.3)	61 (37.2)	61 (41.5)	57 (55.3)	<0.001
Severe	127 (21.9)	39 (23.6)	38 (23.2)	28 (19.0)	22 (21.4)	
Unclear	38 (6.6)	2 (1.2)	17 (10.4)	17 (11.6)	2 (1.9)	
Reported neck pain/stiffness	71 (12.3)	23 (13.9)	22 (13.4)	12 (8.2)	14 (13.6)	0.377
Nausea/vomiting	200 (34.5)	54 (32.7)	65 (39.6)	50 (34.0)	31 (30.1)	0.386
Syncope/loss of consciousness	6 (1.0)	1 (0.6)	4 (2.4)	0 (0.0)	1 (1.0)	0.173
Reported photophobia	36 (6.2)	6 (3.6)	18 (11.0)	5 (3.4)	7 (6.8)	0.016
New limb weakness	13 (2.3)	6 (3.6)	2 (1.2)	1 (0.7)	4 (3.9)	0.162
New limb paresthesia	16 (2.8)	2 (1.2)	9 (5.5)	2 (1.4)	3 (2.9)	0.069
New speech difficulty	6 (1.0)	1 (0.6)	2 (1.2)	2 (1.4)	1 (1.0)	0.917
New visual disturbance	56 (9.7)	23 (13.9)	11 (6.7)	5 (3.4)	17 (16.5)	0.001
Subjective fever or rigors	36 (6.2)	13 (7.9)	11 (6.7)	9 (6.1)	3 (2.9)	0.427
Reported rash	3 (0.5)	2 (1.2)	1 (0.6)	0	0	0.412
Medications taken pre-ED	139 (24.0)	49 (29.7)	45 (27.4)	21 (14.3)	24 (23.3)	0.009
Paracetamol	110 (79.1)	39 (79.6)	37 (82.2)	17 (81.0)	17 (70.8)	0.727
Aspirin	2 (1.4)	2 (4.1)	0	0	0	0.292
NSAID	26 (18.7)	5 (10.2)	10 (22.2)	4 (19.1)	7 (29.2)	0.219
Triptan	3 (2.2)	0	0	0	3 (12.5)	0.002
Tramadol	4 (2.9)	0	2 (4.4)	0	2 (8.3)	0.170
Anti-emetic	1 (0.7)	1 (2.0)	0	0	0	0.604
Physical Examination						
Heart rate > 110 bpm (*n =* 578)	11 (1.9)	2 (1.2)	4 (2.4)	2 (1.4)	3 (2.9)	0.674
SBP > 160 mmHg (*n =* 577)	96 (16.6)	19 (11.5)	30 (18.3)	27 (18.4)	20 (19.4)	0.404
SBP < 90 mmHg (*n =* 577)	1 (0.2)	0	1 (0.6)	1 (0.7)	0	0.639
Temperature > 38 °C (*n =* 576)	8 (1.4)	1 (0.6)	4 (2.4)	3 (2.0)	0	0.314
Glasgow Coma Score	*n =* 458	*n =* 100	*n =* 110	*n =* 147	*n =* 101	
15	448 (97.8)	99 (99.0)	107 (97.3)	144 (97.9)	98 (97.0)	<0.001
13–14	6 (1.3)	0	2 (1.8)	2 (1.4)	2 (2.0)	
<13	4 (0.9)	1 (1.0)	1 (0.9)	1 (0.7)	1 (1.0)	
Rash	7 (1.2)	2 (1.2)	3 (1.8)	0	2 (1.9)	0.425
Confusion	4 (0.7)	0	3 (1.8)	1 (0.7)	0	0.175
Meningism	1 (0.2)	0	0	0	1 (1.0)	0.201
Limited neck flexion	4 (0.7)	2 (1.2)	1 (0.6)	0	1 (1.0)	0.613
New neurological signs	21 (3.6)	8 (4.8)	8 (4.9)	1 (0.7)	4 (3.9)	0.164
New vision defect	6 (1.0)	3 (1.8)	0	0	3 (2.9)	0.051

Data are reported as *n* (%) unless otherwise stated. NSAID: non-steroidal anti-inflammatory drug; SBP: systolic blood pressure. All *p* Values obtained by chi-squared test.

**Table 3:** Investigations and treatment.

Variables	Total (*n =* 579)	KTPH (*n =* 165)	NUH (*n =* 164)	NTFGH (*n =* 147)	SKH (*n =* 103)	*p* Value
Investigations						
CT brain done	191 (33.0)	51 (30.9)	42 (25.6)	52 (35.4)	46 (44.7)	0.011
CT abnormality seen						
SAH Other bleed Neoplasm Infarct Sinusitis Others	40 (20.9)	14 (27.5)	5 (11.9)	13 (25.0)	8 (17.4)	0.150
	1 (2.5)	1 (7.1)	0	0	0	
	10 (25.0)	1 (7.1)	3 (60.0)	5 (38.5)	1 (12.5)	
	1 (2.5)	0	0	1 (7.7)	0	
	10 (25.0)	3 (21.4)	0	3 (23.1)	4 (50.0)	
	13 (32.5)	7 (50.0)	0	3 (23.1)	3 (37.5)	
	5 (12.5)	2 (14.3)	2 (40.0)	1 (7.7)	0	
MRI brain done	11 (1.9)	0	0	2 (1.4)	9 (8.8)	<0.001
CT angiogram brain	5 (0.9)	0	3 (1.8)	2 (1.4)	0	0.208
Treatment						
Medications given in ED	340 (58.7)	88 (53.3)	91 (55.5)	84 (57.1)	77 (74.8)	0.003
Paracetamol	141 (41.5)	32 (36.4)	42 (46.2)	36 (42.9)	31 (40.3)	0.421
NSAID	117 (34.4)	33 (37.5)	32 (35.2)	26 (31.0)	26 (33.8)	0.208
Tramadol	107 (31.5)	36 (40.9)	19 (20.9)	31 (36.9)	21 (27.3)	0.014
Codeine	19 (5.6)	5 (5.7)	7 (7.7)	5 (6.0)	2 (2.6)	0.554
Triptan	3 (0.88)	0	3 (3.3)	0	0	0.041
Ergotamine	3 (0.88)	1 (1.14)	0	2 (2.4)	0	0.294
Prochlorperazine	78 (22.9)	18 (20.5)	31 (34.1)	10 (11.9)	19 (24.7)	0.003
Metoclopramide	59 (17.4)	18 (20.5)	9 (9.9)	24 (28.6)	8 (10.4)	0.001
Ondansetron	7 (2.1)	2 (2.3)	2 (2.2)	1 (1.2)	2 (2.6)	0.929
Required follow-up medications after 30 min	***n =* 340** 49 (14.5)	***n =* 88** 21 (23.9)	***n =* 91** 17 (18.7)	***n =* 84** 1 (1.2)	***n =* 77** 10 (13.3)	<0.001
	49 (14.5)					

Data are reported as *n* (%) unless otherwise stated. CT: computed tomography; ED: emergency department; MRI: magnetic resonance imaging; NSAID: non-steroidal anti-inflammatory drug; SAH: subarachnoid hemorrhage. All *p* Values obtained by chi-squared test.

**Table 4:** Final diagnoses, disposition, and outcome.

Variables	Total (*n =* 579)	KTPH (*n =* 165)	NUH (*n =* 164)	NTFGH (*n =* 147)	SKH (*n =* 103)	*p* Value
Final ED diagnosis						<0.001
Primary benign headache not otherwise specified	257 (44.4)	79 (47.9)	53 (32.3)	94 (64.0)	31 (30.1)	
Migraine	106 (18.3)	26 (15.8)	47 (28.7)	16 (10.9)	17 (16.5)	
Tension headache	59 (10.2)	29 (17.6)	24 (14.6)	4 (2.7)	2 (1.9)	
Sinusitis	25 (4.3)	13 (7.9)	2 (1.2)	3 (2.0)	7 (6.8)	
Hypertension	16 (2.8)	5 (3.0)	1 (0.6)	9 (6.1)	1 (1.0)	
Musculoskeletal headache	12 (2.1)	4 (2.4)	5 (3.1)	2 (1.4)	1 (1.0)	
Viral illness without meningitis	9 (1.6)	4 (2.4)	5 (3.1)	0	0	
Other intracranial hemorrhage	8 (1.4)	0	3 (1.8)	4 (2.7)	1 (1.0)	
Upper respiratory tract infection	8 (1.4)	0	0	8 (5.4)	0	
Ischemic stroke	7 (1.2)	3 (2.0)	1 (0.6)	1 (0.6)	2 (1.9)	
Disposition						<0.001
Home from EDOU	20 (3.5)	7 (4.2)	10 (6.1)	2 (1.4)	1 (1.0)	
Home from ED	422 (72.9)	138 (83.6)	114 (69.5)	110 (74.8)	60 (58.3)	
Admit ward	132 (22.8)	20 (12.1)	37 (22.6)	34 (23.1)	41 (39.8)	
Admit critical care	4 (0.7)	0	2 (1.2)	1 (0.7)	1 (1.0)	
Theatre	1 (0.2)	0	1 (0.6)	0	0	
Discharge diagnosis	***n =* 137**	***n =* 20**	***n =* 40**	***n =* 35**	***n =* 42**	0.011
Migraine	33 (24.3)	7 (35.0)	11 (28.2)	2 (5.7)	13 (31.0)	
Primary benign headache not otherwise specified	24 (17.5)	2 (10.0)	1 (2.5)	11 (31.4)	10 (23.8)	
Tension headache	20 (14.6)	1 (5.0)	7 (17.5)	4 (11.4)	8 (19.1)	
Hypertension	11 (8.0)	1 (5.0)	3 (7.5)	5 (14.3)	2 (4.8)	
Discharge outcome	***n =* 137**	***n =* 20**	***n =* 40**	***n =* 35**	***n =* 42**	0.162 ^
Discharge alive	135 (98.5)	19 (95.0)	40 (100)	34 (97.1)	42 (100)	
Died	2 (1.5)	1 (5.0)	0	1 (2.9)	0	
Length of stay, in days	***n =* 137**	***n =* 20**	***n =* 40**	***n =* 35**	***n =* 42**	0.153 ^#^
Median (IQR)	2 (2–4)	3 (2–4)	3 (2–5)	2 (2–6)	2 (2–3)	
Reattendance within 72 h *	***n =* 442** 6 (1.4)	***n =* 145** 1 (0.7)	***n =* 124** 4 (3.2)	***n =* 112** 0	***n =* 61** 1 (1.6)	0.146 ^

Data are reported as *n* (%) unless otherwise stated. ED: emergency department; EDOU: emergency department observation unit; IQR: interquartile range. All *p* Values obtained by chi-squared test unless otherwise stated. ^ Fisher’s exact test. ^#^ Kruskal–Wallis test. * For patients who were discharged from EDOU or ED.

## Data Availability

The data presented in this study are available on request from the corresponding author. The data are not publicly available due to restrictions by the approving institutional review board.

## Supplementary Materials

The following supporting information can be downloaded at: https://www.mdpi.com/article/10.3390/medicina59071340/s1, Table S1: Attendance and disposition; Table S2: All final ED diagnoses; Table S3: All final hospital diagnosis (for admitted patients).
