# Supplementary material for: Headache in the Emergency Department: A Multicenter Observational Study from Singapore
Source: Medicina (Kaunas). 2023 Jul 21;59(7):1340. doi: 10.3390/medicina59071340 (PMC10384407; doi:10.3390/medicina59071340)
Supplement: Supplementary file 1 [file medicina-59-01340-s001.zip › medicina-2504076-supplementary.pdf]

medicina

MDPI

Supplementary Table S1. Attendance and disposition.

Variables	Total n (%) (n=32425)	KTPH n (%) (n=10081)	NUH n (%) (n=8136)	NTFGH n (%) (n=7913)	SKH n (%) (n=6295)
Admission to ward	10119 (31.2)	2482 (24.6)	2501 (30.7)	2618 (33.1)	2518 (40.0)
Admission to high dependency or intensive care unit	411 (1.3)	128 (1.3)	160 (2.0)	118 (1.5)	5 (0.1)
Transferred to another hospital for care	447 (1.4)	73 (0.7)	201 (2.5)	86 (1.1)	87 (1.4)

Supplementary Table S2. All final ED diagnoses.

Variable	Total n (%) (n=579)	KTPH n (%) (n=165)	NUH n (%) (n=164)	NTFGH n (%) (n=147)	SKH n (%) (n=103)
Final ED diagnosis					
Primary benign headache not otherwise specified	257 (44.4)	79 (47.9)	53 (32.3)	94 (64.0)	31 (30.1)
Migraine	106 (18.3)	26 (15.8)	47 (28.7)	16 (10.9)	17 (16.5)
Tension headache	59 (10.2)	29 (17.6)	24 (14.6)	4 (2.7)	2 (1.9)
Sinusitis	25 (4.3)	13 (7.9)	2 (1.2)	3 (2.0)	7 (6.8)
Hypertension	16 (2.8)	5 (3.0)	1 (0.6)	9 (6.1)	1 (1.0)
Musculoskeletal headache	12 (2.1)	4 (2.4)	5 (3.1)	2 (1.4)	1 (1.0)
Viral illness without meningitis (including dengue)	9 (1.6)	4 (2.4)	5 (3.1)	0	0
Other intracranial hemorrhage	8 (1.4)	0	3 (1.8)	4 (2.7)	1 (1.0)
Upper respiratory tract infection	8 (1.4)	0	0	8 (5.4)	0
Ischemic stroke	7 (1.2)	1 (0.6)	1 (0.6)	3 (2.0)	2 (1.9)
Cluster headache	4 (0.7)	2 (1.2)	1 (0.6)	0	1 (1.0)
Post-traumatic headache	4 (0.7)	1 (0.6)	3 (1.8)	0	0
Trigeminal neuralgia	3 (0.5)	0	1 (0.6)	2 (1.4)	0
Neoplasm	2 (0.3)	0	1 (0.6)	1 (0.7)	0
Viral meningitis	2 (0.3)	0	2 (1.2)	0	0
Subarachnoid hemorrhage	1 (0.2)	1 (0.6)	0	0	0
Post-coital headache	1 (0.2)	0	0	1 (0.7)	0
Analgesia overuse	1 (0.2)	0	1 (0.6)	0	0
Others	4 (0.7)	0	2 (1.2)	0	2 (1.9)
Unclear cause	50 (8.6)	0	12 (7.3)	0	38 (36.9)

Supplementary Table S3. All final hospital diagnosis (for admitted patients).

Variables	Total n (%) (n=137)	KTPH n (%) (n=20)	NUH n (%) (n=40)	NTFGH n (%) (n=35)	SKH n (%) (n=42)
Final hospital diagnosis					
Migraine	33 (24.1)	7 (35.0)	11 (27.5)	2 (5.7)	13 (31.0)
Primary benign headache not otherwise specified	24 (17.5)	2 (10.0)	1 (2.5)	11 (31.4)	10 (23.8)
Tension	20 (14.6)	1 (5.0)	7 (17.5)	4 (11.4)	8 (19.1)
Hypertension	11 (8.0)	1 (5.0)	3 (7.5)	5 (14.3)	2 (4.8)
Other intracranial hemorrhage	9 (6.6)	0	4 (10.0)	4 (11.4)	1 (2.4)
Musculoskeletal headache	9 (6.6)	1 (5.0)	7 (17.5)	4 (11.4)	8 (19.1)
Sinusitis	4 (2.9)	0	0	1 (2.9)	3 (7.1)
Neoplasm	3 (2.2)	0	2 (5.0)	1 (2.9)	0
Viral illness without meningitis	3 (2.2)	1 (5.0)	1 (2.5)	1 (2.9)	0
Viral meningitis	2 (1.5)	0	2 (5.0)	0	0
Ischemic stroke	2 (1.5)	0	0	2 (5.7)	0
Cluster headache	2 (1.5)	1 (5.0)	0	0	1 (2.4)
Subarachnoid hemorrhage	1 (0.7)	1 (5.0)	0	0	0
Glaucoma	1 (0.7)	0	1 (2.5)	0	0
Post-traumatic headache	1 (0.7)	0	1 (2.5)	0	0
Others	11 (8.0)	5 (25.0)	4 (10.0)	1 (2.9)	1 (2.4)
Unclear	1 (0.7)	0	1 (2.5)	0	0
